# Case of “Glanders” in the Human Subject

**Published:** 1845-12

**Authors:** C. Smallwood

**Affiliations:** St. Martin


*Case of "Glanders" in the Human Subject.—(Equinia.)*

BY C. SMALLWOOD, M. D., ST. MARTIN.

*History.*—Louis H., married, æt. 42, farmer, of spare habit, given to drink rather freely of spirituous liquors, otherwise healthy; was taken ill on Saturday, 20th April, 1844, with a pain in the head and back of the neck, which prevents him from sleeping.

*Present State.*—Applied this day, 22nd, at 5, p. m., complains of headache, pain in the back of the neck, and limbs; the pain at the back of the neck increased by motion; skin hot; a slight redness and tumefaction of the right eye-lid; pulse 90; tongue moist, covered with a brownish fur; slight thirst; loss of appetite; bowels natural; urine high coloured.

℞ Hyd. Submur.
Pulv. Antimon. *aa* gr. v. Fiat. Pulv. S. S.

℞ Magnes. Sulphat. ℥i.

Acid. Sulphur. Dilut. ʒij.

Potas. Nitrat. ʒi.

Aquæ. Menth. Piperit. ℥vij. M. Hujus. mist. sumat. cochl. quart. omni hora. donec venter rite solutus fuerit.

℞ Liq. Plumb. Acetat. Dilut. Ol. Lint. quadruplicat. hoc liquore frigido madefact. partibus inflam. applicent., et sæpissime renoventur.

24th.—His wife applied this day; states the pain in the neck and limbs not alleviated; headache diminished; reports the tumefaction of the eye-lid increased; soreness of the throat; bowels have been freely opened, (by medicines ordered); fæces dark coloured and offensive.

Repetant Mist. et Lotio.

25th. —Visited him this day, at 4, p. m., (about a league distant), the redness and swelling increased, so as to completely close the eye-lid, with great heat; redness does not disappear on pressure; great estlessness; skin hot; pulse100; difficult deglutition; pharynx and tonsils trumefied and red; increased secretion from the nostrils and saliva; breath very offensive; tongue furred; bowels loose; fæces dark and very offensive.

℞ Hyd. Submur. gr. xxiv.
Pulv. Opii gr. iij.—Fiat. Massa in Pillul. xij. dividend. Sumatur una omni hora.

*Midnight.*—Complains of intense heat of the head, neck and throat, so much so that he is constantly calling to have cold water applied; a secretion of viscid mucus, of a yellow colour, from his nostrils and throat; the tumefaction extended to both eye-lids; increased difficulty in deglutition and respiration; skin moist; pulse 110; occasional delirium; dejections dark, liquid, and offensive; urine high coloured.

℞ Tinct. Hyosciami, ʒi.

Mist. Camph. ℥i.—Ft. Haustus S. S.

26th.—1, p. m., swelling of the eye-lid increased so as entirely to prevent vision; skin diminished in temperature; complete inability to swallow; increased secretion from the nose and throat, of a dark colour, and viscid; the swelling has assumed a lurid hue: delirium; tongue coated with a dark fur; vibices; pulse 120, and small; involuntary dejections and very offensive: (endeavoured to force down some wine but could not;) a number of pustules appeared this morning on the legs and body, and two on the face, as large as those in variola, containing a watery fluid, of a dark red colour; respiration laborious; constant muttering, and picking at the bed-clothes; urine fœtid and dark; skin bathed in perspiration. Ordered wine to be given frequently, and to gargle with wine and water.

27th.—8, a. m., evidently sinking; scarcely able to rouse him; respiration still laborious, has swallowed a few spoonfuls of broth; I forced down some wine; pustules shrunk and livid; secretion from the nose and throat copious and very offensive; cannot swallow. Left a mixture of Ammonia and Camphor, of which, he did not take but one dose.

28th.—Died at 6, a. m.; the friends would not consent to a *post mortem* examination.

*Remarks.*—I did not, until my visit to his house on the 25th, (the third day after he applied in person), suspect the true nature of the disease, but from the train of alarming symptoms then present, I made a more careful inquiry, and it was with some difficulty I succeeded in ascertaining the facts of the case, which left no doubt as to its real nature. The mare from which the contagion was propagated, died shortly afterwards with confirmed glanders. It would appear that my patient was administering a medicinal drink to the animal, 2 or 3 days previous to his illness, and that she snorted some of the drink into his face, to which he paid no attention, and thought so very lightly of it, that he did not even wash his face for some time afterwards; he was assisted in the operation of *drenching* the animal, by his son, who escaped the disease. The mare, during the time she was in his possession, (which was only a short time,) did not communicate the disease to any other of his cattle; she was sold twice afterwards, and died in about 20 days from the death of my patient.

The *diagnostic* marks of the disease, as far as my observation goes, consists of, 1st. The increased secretion of the nose and throat. 2nd, The intense sensation of heat in the head, neck, and throat, (it was most distressing to hear the poor fellow crying out for cold water to be thrown over him). And 3rd. To the heat suceeeds a very copious fœtid discharge. The inflammatory symptoms having given way to the typhoid, and, I may add, the pustular eruption.

At a future time I shall recur to this subject.

*St. Martin, Isle Jesus, Oct.* 25, 1845.

[The exceeding rarity of this disease, in this country, (this is the only case of which we have heard), very naturally points to the inquiry, whether professional men, located in other parts of the Province, have met with similar cases. We are exceedingly obliged to Dr. Smallwood, for the above communication, for it has, at least, tended to remove one erroneous impression under which we laboured, that glanders in the human subject was unknown in this Province: not by any means that the disease has not been well recognized in veterinary practice, but that from the influence of climate, its virus had become mitigated, if not destroyed; for we can scarcely imagine that occasions for inoculation are not as frequent here as in Europe.—Eds.]

